# Supplementary material for: The Systems Biology Research Tool: evolvable open-source software
Source: BMC Syst Biol. 2008 Jun 29;2:55. doi: 10.1186/1752-0509-2-55 (PMC2446383; doi:10.1186/1752-0509-2-55)
Supplement: Additional file 1 — SBRT Archive. An archive of the current version of the Systems Biology Research Tool. [file 1752-0509-2-55-S1.zip › sbrt-1.4.0/doc/developers_guide/api/sbrt/kernel/math/graph_theory/Path.html]

Path


|  |  |  |
| --- | --- | --- |
| **Overview** \| **Package** \| **Class** \| **Use** \| **Tree** \| **Deprecated** \| **Index** \| **Help** | |  |
| **PREV CLASS**   NEXT CLASS | **FRAMES**    **NO FRAMES**     **All Classes** |  |
| SUMMARY: NESTED \| FIELD \| CONSTR \| METHOD | DETAIL: FIELD \| CONSTR \| METHOD |  |


---


## sbrt.kernel.math.graph\_theory Class Path<N>

```
java.lang.Object
  sbrt.kernel.math.graph_theory.Path<N>
```

**Type Parameters:**: `N` - the node type.

---

``` public class Path<N> extends java.lang.Object ```

This class can be used for representing paths in a graph
or network. A path is a sequence
{x_0_, x_1_, ..., x_n-1_}
such that (x_0_, x_1_), (x_1_, x_2_),
..., (x_n-2_, x_n-1_) are
edges of a graph, where n is the total number of nodes in the path.

Instances of this class are immutable.

**Author:**
:   This class was written and documented by
    Jeremiah Wright while in the Wagner lab.

---

**Constructor Summary**

`Path(java.util.Collection<? extends N> path)`
  
          Constructs a path containing the nodes from the provided
collection, in the order they are returned from the
collection's iterator.

`Path(T[] path)`
  
          Constructs a path containing the nodes from the provided
array, in the order they occur starting from index 0.


| **Method Summary** | |
| --- | --- |
| `boolean` | `contains(java.lang.Object node)`             Returns true if this path contains the specified node. |
| `boolean` | `equals(java.lang.Object obj)`             Compares the specified object with this path for equality. |
| `N` | `get(int index)`             Returns the node at the specified position in this path. |
| `int` | `hashCode()`             Returns a hash code based on the nodes contained in this path. |
| `int` | `indexOf(java.lang.Object node)`             Returns the index in this path of the first occurrence of the specified node, or -1 if this path does not contain that node. |
| `java.util.Iterator<N>` | `iterator()`             Returns an iterator over the nodes in this path in proper sequence. |
| `int` | `nodes()`             Returns the number of nodes in this path. |
| `N[]` | `toArray()`             Returns an array representation of this path. |
| `java.util.List<N>` | `toList()`             Returns a list representation of this path. |
| `java.lang.String` | `toString()`             Returns a string representation of this path. |

**Methods inherited from class java.lang.Object**

`clone, finalize, getClass, notify, notifyAll, wait, wait, wait`

**Constructor Detail**

### Path

```
public Path(T[] path)
```

:   Constructs a path containing the nodes from the provided
    array, in the order they occur starting from index 0.

    **Type Parameters:**: `T` - a type extending the primary node type. **Parameters:**: `path` - the array whose elements will represent nodes in this path. **Throws:**: `java.lang.NullPointerException` - if the provided array is null or if a node contained within that array is `null`.

---


### Path

```
public Path(java.util.Collection<? extends N> path)
```

:   Constructs a path containing the nodes from the provided
    collection, in the order they are returned from the
    collection's iterator.

    **Parameters:**: `path` - the collection whose elements will represent nodes in this path. **Throws:**: `java.lang.NullPointerException` - if the provided collection is `null` or if a node contained within that collection is `null`.


**Method Detail**

### nodes

```
public int nodes()
```

:   Returns the number of nodes in this path.

    :   **Returns:**: the number of nodes in this path.

---


### get

```
public N get(int index)
```

:   Returns the node at the specified position in this path.

    :   **Parameters:**: `index` - index of node to return. **Returns:**: the node at the specified position in this path. **Throws:**: `java.lang.IndexOutOfBoundsException` - if index is out of range (index < 0 || index >= nodes()).

---


### indexOf

```
public int indexOf(java.lang.Object node)
```

:   Returns the index in this path of the first occurrence of the
    specified node, or -1 if this path does not contain that node.

    :   **Parameters:**: `node` - node to search for. **Returns:**: the index in this path of the first occurrence of the specified node, or -1 if this path does not contain that node.

---


### contains

```
public boolean contains(java.lang.Object node)
```

:   Returns true if this path contains the specified node.

    :   **Parameters:**: `node` - node whose presence in this Path is to be tested. **Returns:**: `true` if the specified node is present; `false` otherwise.

---


### iterator

```
public java.util.Iterator<N> iterator()
```

:   Returns an iterator over the nodes in this path in
    proper sequence.

    :   **Returns:**: an iterator over the nodes in this path in proper sequence.

---


### toArray

```
public N[] toArray()
```

:   Returns an array representation of this path. Nodes in
    this path become elements in the returned array. The
    nodes occur in the same order as the do in this path,
    starting from index 0.

    :   **Returns:**: an array representation of this path.

---


### toList

```
public java.util.List<N> toList()
```

:   Returns a list representation of this path. Nodes in this
    path become elements in the returned list. The nodes occur
    in the same order as they do in the path.

    :   **Returns:**: a list representation of this path.

---


### equals

```
public boolean equals(java.lang.Object obj)
```

:   Compares the specified object with this path for equality.
    Returns true if and only if the specified object is also a
    path, both paths have the same size, and all corresponding
    pairs of nodes in the two paths are equal. In other words,
    two paths are defined to be equal if they contain the same
    nodes in the same order.

    :   **Overrides:**: `equals` in class `java.lang.Object`
    :   **Parameters:**: `obj` - the object with which to compare. **Returns:**: true if the specified object is equal to this path.

---


### hashCode

```
public int hashCode()
```

:   Returns a hash code based on the nodes contained in this path.

    :   **Overrides:**: `hashCode` in class `java.lang.Object`
    :   **Returns:**: a content-based hash code

---


### toString

```
public java.lang.String toString()
```

:   Returns a string representation of this path.

    :   **Overrides:**: `toString` in class `java.lang.Object`
    :   **Returns:**: a string representation of this path.


---


|  |  |  |
| --- | --- | --- |
| **Overview** \| **Package** \| **Class** \| **Use** \| **Tree** \| **Deprecated** \| **Index** \| **Help** | |  |
| **PREV CLASS**   NEXT CLASS | **FRAMES**    **NO FRAMES**     **All Classes** |  |
| SUMMARY: NESTED \| FIELD \| CONSTR \| METHOD | DETAIL: FIELD \| CONSTR \| METHOD |  |


---
